# Exploring *Plasmodium falciparum Var* Gene Expression to Assess Host Selection Pressure on Parasites During Infancy

**DOI:** 10.3389/fimmu.2019.02328

**Published:** 2019-10-09

**Authors:** Cheryl A. Kivisi, Michelle Muthui, Martin Hunt, Greg Fegan, Thomas Dan Otto, George Githinji, George M. Warimwe, Richard Rance, Kevin Marsh, Peter C. Bull, Abdirahman I. Abdi

**Affiliations:** ^1^KEMRI Wellcome Trust Research Programme, Kilifi, Kenya; ^2^Pwani University Biosciences Research Centre, Pwani University, Kilifi, Kenya; ^3^Department of Biological Sciences, Pwani University, Kilifi, Kenya; ^4^Wellcome Sanger Institute, Cambridge, United Kingdom; ^5^Centre for Tropical Medicine and Global Health, University of Oxford, Oxford, United Kingdom

**Keywords:** malaria, maternal antibodies, var gene expression, age, infants

## Abstract

In sub-Saharan Africa, children below 5 years bear the greatest burden of severe malaria because they lack naturally acquired immunity that develops following repeated exposure to infections by *Plasmodium falciparum*. Antibodies to the surface of *P. falciparum* infected erythrocytes (IE) play an important role in this immunity. In children under the age of 6 months, relative protection from severe malaria is observed and this is thought to be partly due to trans-placental acquired protective maternal antibodies. However, the protective effect of maternal antibodies has not been fully established, especially the role of antibodies to variant surface antigens (VSA) expressed on IE. Here, we assessed the immune pressure on parasites infecting infants using markers associated with the acquisition of naturally acquired immunity to surface antigens. We hypothesized that, if maternal antibodies to VSA imposed a selection pressure on parasites, then the expression of a relatively conserved subset of *var* genes called group A *var* genes in infants should change with waning maternal antibodies. To test this, we compared their expression in parasites from children between 0 and 12 months and above 12 months of age. The transcript quantity and the proportional expression of group A *var* subgroup, including those containing domain cassette 13, were positively associated with age during the first year of life, which contrasts with above 12 months. This was accompanied by a decline in infected erythrocyte surface antibodies and an increase in parasitemia during this period. The observed increase in group A *var* gene expression with age in the first year of life, when the maternal antibodies are waning and before acquisition of naturally acquired antibodies with repeated exposure, is consistent with the idea that maternally acquired antibodies impose a selection pressure on parasites that infect infants and may play a role in protecting these infants against severe malaria.

## Introduction

In many malaria endemic regions, incidence of severe malaria is rare in children younger than 6 months (1, 2) suggesting a potential role of maternal antibodies in protection from malaria. In a cross sectional survey in Benin and Gambia, it was reported that the mean parasite densities among infants younger than 1 year was significantly lower than in children aged between 1 and 9 years (3). Several studies have explored the role of antibodies in this phenomenon (4–8). However, because of the difficulty in conclusively linking antibody carriage to clinical protection, the mechanism of protection and the targets of the maternal antibodies remain unclear.

An alternative approach to explore the potential role of host antibodies, is to explore the parasites that cause infections in infants and seek parasite markers that will act as a read-out of *in vivo* antibody selection on the infecting parasites populations. The *Pf*EMP1 family of antigens that are expressed on the surface of parasite infected erythrocytes provide a potentially useful marker for this purpose. *Pf*EMP1 is a highly diverse family of multi-domain parasite antigens that are inserted into the infected erythrocyte surface, encoded by a family of about 60 *var* genes in every genome (9, 10). *Pf*EMP1 mediates the binding of Infected Erythrocytes (IE) to vascular endothelial cells, allowing the parasite to sequester in the microvasculature of the organs and avoid clearance by the spleen (11). Therefore, through sequestration, *Pf*EMP1 supports parasite growth and plays an important role in the pathogenesis of malaria.

Despite their overall high level of diversity, parasites encode subsets of *Pf*EMP1 antigens that are relatively conserved. A subset of *Pf*EMP1 referred to as group A and those containing commonly occurring domain arrangements called domain cassettes 8 and 13 (DC8 and DC13) are thought to play a role in the pathogenesis of malaria (12, 13). *In vitro* studies have shown that these subsets of *Pf*EMP1 mediate strong binding to endothelial cells (14) and to non-infected erythrocytes in a process referred to as rosetting (15, 16). Therefore, through their cytoadhesive properties, they have been hypothesized to support parasite growth, increasing the risk of severe malaria. Further, these *var* subsets are more commonly expressed in parasites from children with low host immunity and those with severe malaria (17, 18). Although most studies on clinical *P. falciparum* isolates have found that host age is negatively correlated with expression of group A and DC8 *var* genes (18–20), these studies have not considered *var* expression in parasites sampled from children with malaria below 12 months, possibly due to the extreme rarity of *P. falciparum* infection in children in this age group (21). In the study described here, we aimed to overcome the rarity of parasites sampled from infants by making use of a large collection of parasite isolates that have been collected over a 16-year period. We hypothesized that if maternal antibodies are important in the protection of children from malaria in early life, there will be a positive association between the expression of group A *var* genes in parasites and the age of the children in the first 12 months of life, as maternal antibodies wane.

## Materials and Methods

### Study Site, Sample Collection, and Ethics

The study was carried out at Kilifi County which is situated on the Kenyan coast. Parasite isolates and plasma samples collected between 1994 and 2012, from *Plasmodium falciparum* positive pediatric admissions and longitudinal cohort children, were used for the study. Ethical approval was obtained from the Kenya Medical Research Institute Scientific and Ethics Review Unit (KEMRI/SERU) under the protocol; KEMRI/SERU/3149, and informed consent was obtained from the parents/guardians of the children.

### *Var* Expression Analysis

RNA was obtained from TRIzol™ reagent (Invitrogen, catalog number 15596026) preserved *P. falciparum* positive venous blood samples, obtained from the children recruited for the study. RNA was extracted using a Chloroform method (19) and cDNA was synthesized using the Superscript III kit (Invitrogen, catalog number 18091050) following the manufacturer's protocol. *Var* gene expression analysis was carried out through (a) PCR amplification of a conserved region of the *var* genes (expressed sequence tags) and sequencing using capillary and 454 platforms, and (b) quantitative real-time PCR as described below.

- *** Expressed sequence Tag (EST) sequencing***
  Using degenerate AF and BR primers (22), EST consisting of the conserved DBLα region of the *var* genes were amplified from the cDNA of each isolate by PCR. The PCR product was cleaned and sequenced as described below.
  - ***454 sequencing and sequence assembly***
    To enable sequencing, a multiple identifier (MID) tag was ligated onto PCR products from each isolate. A total of 48 MID tags were available allowing for pooling of 48 different isolates before sequencing using the 454 GS-FLX 3 Titanium technology using the standard protocol (Roche). For each pool, sequencing success was determined using the amount of data and length distribution of reads at the end of the sequencing run. Pools that did not meet these criteria were resequenced.
    Reads from the data were partitioned using their MID and pool identity. Reads shorter than 300 bp were excluded from further analysis. The following methods were used independently for each set of reads. The reads were assembled with cap3 (version date 12/21/07) using the default settings (23). The resulting contigs were clustered by putting contigs with BLAST (version 2.2.25) matches of at least 98% identity and 95% match length of the longest sequence into the same cluster. Each cluster of contigs was assembled using cap3 with the default settings (23). A ruby program bio-dbl-classifier was used for classification of each of the assembled contigs into their respective DBLα subgroup as described in Bull et al. (24). Concurrently, the level of expression of each assembled contig was determined by mapping all the reads from that isolate to the assembled consensus contig sequences from the same isolate at a 95% identity. The number of reads per contig, DBLα classification file and the total number of reads for each isolate were then used to determine the proportion of expression of each of the different DBLα subgroups in each isolate using STATA as follows e.g., for each isolate,
    (1) $$\begin{array}{l} \%\;expression\;of\;Group\;A-like\\ \;=\frac{no\;of\;contigs\;classified\;as\;group\;A\;-\;like\;}{total\;no\;of\;reads\;}\times 100 \end{array}$$
  - ***EST sequencing by capillary***
    For 237 samples, the capillary sequencing platform was used to sequence the amplified EST as described (18, 24). The *var* expression data published in these studies are included in this study.
- ***Var transcript quantification using RT-PCR***
  Real-time PCR data was obtained as described (17, 25, 26). Four primers targeting DC8 (named dc8-1, dc8-2, dc8-3, dc8-4), one primer targeting DC13 (dc13) and two primers targeting the majority of group A *var* genes (gpA1 and gpA2) were used in real-time PCR analysis (Table S1). We also used two primers, b1 and c2, targeting group B and C *var* genes, respectively (27) (Table S1). Two housekeeping genes, Seryl tRNA synthetase and Fructose bisphosphate aldolase (20, 28, 29) were used for relative quantification of the expressed *var* genes. The PCR reaction and cycling conditions were carried out as described in Lavstsen et al. (20) with the Applied Biosystems 7500 Real-time PCR system. We set the cycle threshold (Ct) at 0.025. Controls with no template were included at the end of each batch of 22 samples per primer and the melt-curves analyzed for non-specific amplification. Genomic DNA from the IT4 laboratory parasite line at 10 ng/μl was used as a standard sample in all plates. The var gene “transcript quantity” was determined relative to the mean transcript of the two housekeeping genes, Seryl tRNA synthetase and Fructose biphosphate aldolase as described (20). For each test primer, the Δct for both the test samples and the standard genomic DNA was calculated and used to generate the ΔΔct value which was then transformed to arbitrary transcript unit (Tu_s_) using the formula [Tu_s_ = 2^(5−ΔΔct)^]. However, we also estimated “proportional expression” of the transcripts within each sample. When calculating proportional expression from qPCR (described under “statistical analysis” below), Tu_s_ calculated as described in Lavstsen et al. (20) i.e., 2^(5−Δct)^ were used. We assigned a zero Tu_s_ value if a reaction did not result in detectable amplification after 40 cycles of amplification, i.e., if the Ct value was undetermined.

### Flow Cytometry

We had a measure of antibodies to infected erythrocytes (αIE) for 215 children from a previously published work (18), of which 27 were aged between 0 and 12 months and the rest were older than 12 months. To boost the αIE data for the children aged between 0 and 12 months, we measured αIE levels for 69 children who had available archived acute plasma samples. The antibody level that each child had at the time of parasite sampling was determined using Flow cytometry against one *P. falciparum* clinical isolate matured *ex vivo* to the trophozoite stage and cryopreserved in liquid nitrogen as described in Warimwe et al. (18). The cryopreserved trophozoites were thawed as previously described (30), erythrocyte pellet volume estimated, parasitemia determined by microscopy and diluted to 1% using fresh uninfected erythrocytes. The cells were resuspended in 0.5% bovine serum albumin in 1% phosphate buffered saline (0.5% BSA/PBS) such that 11.5 μl of the mixture contained 0.5 μl of the cells. The reaction mixture was stained with Ethidium bromide at a final concentration of 10 μg/ml and 11.5 μl of the mixture per well was aliquoted into U-bottom 96-well plates (Falcon®, catalog number BD 353077). One microliter of each child's acute plasma was pipetted into separate wells and the mixture incubated for 30 min at room temperature. Following three washes with 0.5% BSA/PBS, the cells were incubated in the dark for 30 min at room temperature with 50 μl/well of fluorescein isothiocynate (FITC)-conjugated sheep anti-human IgG (Binding Site, catalog number AF004.X) diluted 1:50 in 0.5% BSA/PBS. Following three washes, the cells were diluted 10-fold in 0.5% BSA/PBS and at least 1000 IE acquired on a BD FACs Canto II cytometer (BD Biosciences). Plasma from four non-exposed European donors and one from a semi-immune Kilifi adult were included in each plate and used as negative and positive controls, respectively.

Data analysis was done using FlowJo v10 software (TreeStar Inc.). Following initial gating to remove singlets, IE were distinguished from uninfected erythrocytes on the basis of their Ethidum Bromide staining. Next, median fluorescence intensity on the FITC channel from each isolate was obtained by subtracting the signal of the uninfected from the infected erythrocytes signal (Figure S1). To cater for non-specific binding, the mean median fluorescent intensity of the four non-exposed sera on each plate was subtracted from the median florescence intensities of each child's sample.

### Statistical Analysis

The median transcript units from quantitative real time PCR were calculated as follows; DC8 median from four primers used (dc8-1, dc8-2, dc8-3 and dc8-4) and group A median from two primers (gpA1 and gpA2). The proportion of transcripts for each *var* group was then calculated from the sum of transcript (sum of DC8 median, Group A median, DC13, B1, and C2) e.g.,

(2) $$\begin{array}{r} DC8\;proportion=\frac{DC8\;median}{sum\;of\;transcript} \end{array}$$

Spearman's rank correlation was used to determine the strength of the correlations between different variables (e.g., *var* gene expression with host age and host αIE**)**. Scatter plots were used to visualize the direction of correlations between *var* expression and various variables including host age and αIE levels. All analysis was done using Stata V13.

## Results

We used an expressed sequence tag approach (EST) to broadly look at the proportional expression of different subsets of *var* genes. In addition, we used a real time PCR approach using primers designed to detect various subsets of *var* genes. The number of isolates whose *var* expression was analyzed using the two approaches and more information about the primers used is summarized in Table S1.

### Parasite Group A *Var* Gene Expression Increases With Age in the First Year of Life

**Figure 1:**
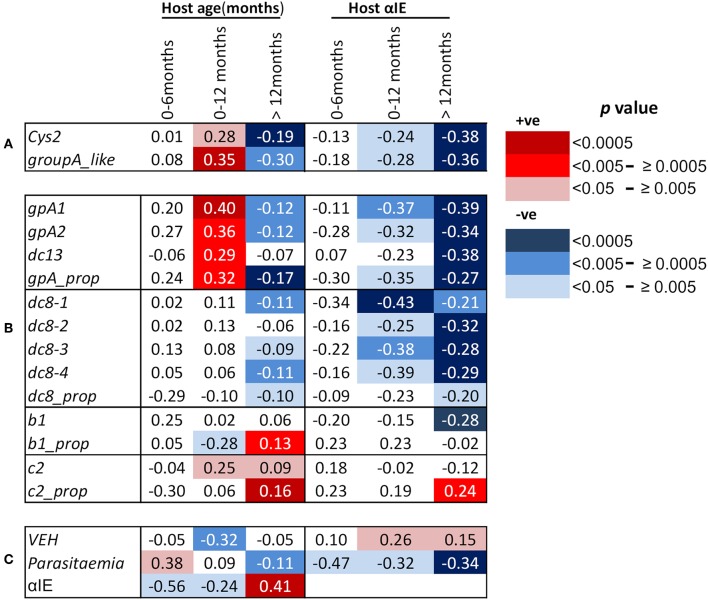
Correlations between host age in months, host antibodies to infected erythrocytes (αIE) and *var* gene expression of the infecting parasites. Shown are Spearman's rank correlation coefficient (rho) for the associations between host age, host antibodies to IE (αIE), and expression of *var* genes of the infecting parasites in children aged 0–6, 0–12, and above 12 months. Red background shading indicate positive correlation while blue shading indicate a negative correlation. Increasing color intensity indicates increasing statistical significance of the correlation as indicated in the key [*p* < 0.00005 (darkest), *p* < 0.05 (lightest)]. **(A)** Associations of age of the children and their αIE levels with expression of cys2 and group A-like *var* genes, **(B)** associations of age of the children and their αIE levels with *var* transcript quantity obtained with primers targeting (i) group A *var* genes (gpA1, gpA2, dc13) and their proportion relative to the sum of the *var* transcript measured (gpA_prop), (ii)DC8 *var* genes (dc8-1, dc8-2, dc8-3, and dc8-4) and their proportional expression (dc8_prop), (iii) group B *var* genes (b1) and their proportional expression (b1_prop), and (iv) group C *var* genes (c2) and their proportional expression (c2_prop), **(C)** associations of age of the children and their αIE levels with *var* gene expression homogeneity (VEH). Also shown is the associations of parasitemia with age and αIE levels of the children and the relationship between αIE and age of the children. More details about the primers can be found in Table S1.

We and others have previously reported negative correlations between age and the expression of group A-like *var* genes measured using the EST approach (15, 18, 19, 31). A similar observation was made when expression of group A and DC8 was quantified by qPCR (17). In this study, we looked at the correlation between age and *var* gene expression in the first year of life and thereafter. We found that the expression of cys2 and group A-like *var* genes, as determined from EST, was positively correlated with host age in the first 12 months (cys2; *p* = 0.005, group A-like; *p* = 0.0004, Figure 1A, Figures S2A,B). Similarly, positive correlations were found with the transcript quantity of group A *var* genes determined with both gpA1 and gpA2 primers (Table S1) designed to target broad group A *var* genes (gpA1; *p* = 0.00007, gpA2; *p* = 0.003, Figure 1B, Figures S2C,D). The relationships between the expression of group A *var* genes and host age remained significant after Bonferroni correction for 10 multiple comparisons (*p* = 0.0007, and *p* = 0.03 for gpA1, and gpA2, respectively). In the first year of life, expression of DC13 was positively associated with host age (*p* < 0.005, Figure 1B, Figure S2E) though not significant after correction for 10 multiple comparison (*p* = 0.05). The expression of group B *var* genes as determined with b1 (Figure 1B, Figure S2J) and DC8 primers did not show significant association with host age in the first year of life (Figure 1B, Figures S2F,G). Unexpectedly, expression of group C *var* genes (c2) showed positive associations with host age (*p* < 0.02, Figure 1B, Figure S2K).

We hypothesized that the positive correlation between the expression of group A *var* genes and host age in the first year of life was due to the decay of maternal antibodies. If this were the case, an opposite effect would be expected in children above 12 months as they become exposed to malaria and make antibodies. In support of this, the transcript quantity of group A *var* genes (except DC13) in children over 12 months was negatively associated with host age (group A-like; *p* = 0.004, gpA1; *p* = 0.0016, gpA2; *p* = 0.0004, Figures 1A,B, Figures S2A–D). The relationship between host age and the transcript quantity of DC8 *var* genes, as expected, was in the negative direction (Figure 1B, Figures S2F–I).

In an alternative approach, the proportion of group A, B, C, and DC8 *var* gene transcript was calculated from the estimated overall transcript quantity of group A, DC13, DC8, group B, and C (17). In this analysis, only a subset of 654 observations that had expression data for all the *var* subsets measured and age were included (Table S1). The proportion of group B *var* genes (b1_prop) was negatively associated with host age in the first year of life (*p* = 0.01, Figure 1B), and positively associated with host age in children older than 12 months (*p* = 0.002, Figure 1B). In contrast, the proportional expression of group A *var* genes (gpA_prop) was positively associated with host age in the first year of life (*p* = 0.004, Figure 1B) and negatively associated with host age in children older than 12 months (*p* = 0.00004, Figure 1B). DC8 proportional expression association with host age in the first year of life was not significant (Figure 1B) but showed weak significant association after 12 months of life (Figure 1B).

Group C proportional expression was not associated with age in the first 12 months but was positively associated with age after 12 months (*p* = 0.0001, Figure 1B).

### Antibodies to Infected Erythrocyte Surface Antigens (αIE) Showed Negative and Positive Associations With Host Age in Children Up to 6 Months and Above 12 Months, Respectively

In malaria endemic regions, it is expected that there is a positive correlation between host age and antibody levels (18). For this study, due to the relatively rapid decay in maternal antibodies during the first 6 months of life and slow simultaneous acquisition of antibodies following exposure to malaria, we hypothesized that overall there will be a decline in host antibodies with age in the first year, particularly in the first 6 months. Consistent with waning maternal antibodies, the αIE levels was negatively correlated with host age, especially in the first 6 months (*p* = 0.008, Figure 1C). In contrast, positive correlation between host age and αIE in children older than 12 months was observed (*p* < 0.00001, Figure 1C).

### αIE Were Negatively Associated With Expression of Group A and DC8 *Var* Gene Subsets

As expected, we found significant negative correlations between group A expression (both transcript quantity and proportional expression) and αIE in both 0–12 months and above 12 months old children (Figures 1A,B). Similar correlation was also observed between DC8 expression and αIE (Figure 1B). The correlation between group B (b1) transcript quantity and αIE was in the negative direction but significant only in the children older than 12 months (Figure 1B) while group C (c2) transcript quantity showed no association with αIE (Figure 1B). The proportional expression of group B (b1_prop) and C (c2_prop) were also not significantly correlated with αIE in the children 0–12 months old, however, group C proportional expression showed significant positive correlation with αIE (Figure 1B). This is in contrast to group A(gpA_prop) and DC8 (dc8_prop) proportional expression which were in the negative direction (Figure 1B). The correlation between *var* gene expression and αIE in children aged 0–6 months was consistent with that of 0–12 months, although the association was not reaching statistical significance, most likely due to small sample size (Figures 1A,B).

### *Var* Expression Homogeneity (VEH) Was Negatively Associated With Host Age and Positively With Host Immunity (αIE) in the First Year of Life

Previously, we observed the homogeneity of *var* expression to increase with increasing host immunity. That is, the *var* gene expression in parasites from naïve children was more heterogeneous compared to semi-immune children such as asymptomatics (32). In this study, we observed, in the first year of life, VEH decreased with declining αIE and increasing age as demonstrated by the positive and negative association with host αIE and age, respectively (αIE; *p* = 0.03, age; *p* = 0.001, Figure 1C).

### Parasitemia Increased With Declining αIE and Increasing Host Age in the First Year of Life

We finally examined the potential role of αIE in controlling parasitemia in the first year of life and after (Figure 1C). As shown in Figure 1C, parasitemia showed positive association with host age in the first 6 months of life (*p* = 0.03, Figure 1C). During this period, parasitemia also increased with declining αIE as shown by the negative correlation with αIE (*p* = 0.03, Figure 1C). In contrast, parasitemia decreased with increasing host age and αIE after 12 months of life (age; *p* = 0.002, αIE; *p* < 0.0001, Figure 1C) when the children are expected to acquire αIE with time as a result of continuous exposure to malaria. This result reinforces a role for maternal αIE in limiting *in vivo* parasite expansion and severe malaria during infancy.

## Discussion

This study set out to investigate the relationship between the expression of *var* genes that have been associated with severe malaria and host age in children between 0 and 12 months, the age during which maternal immunity declines. We hypothesized that there will be a positive correlation between the expression of group A *var* genes and host age in children 0–12 months old after which the relationship will change to a negative correlation. As expected, DC8, DC13 and group A *var* genes showed positive associations with host age in children 0–12 months old.

The simplest explanation for these changes in *var* expression patterns is that they reflect the decay of maternal antibodies with increasing age in children between 0 and 12 months old, leading to increased survival of parasites expressing group A *var* genes. This allows parasites expressing group A *var* genes to dominate the infection. Subsequent acquisition of antibodies against these *var* genes results as children gain exposure to infection, consequently leading to a decline in group A-like *var* gene expression levels in older children (>12 months). Our result is consistent with the above explanation as αIE levels declined in the first 6 months of age, a period of rapid decline of maternal antibodies as observed in other studies (4, 5).

As expected the αIE showed negative association with both host age and *var* expression in the children between 0 and 12 months old and the subset <6 months of age. This contrasts with the positive relationship between host age and αIE in children older than 12 months (Figure 1). One caveat of the αIE data for the children 0–12 months is that the αIE levels were measured against one clinical isolate but in a previous study in which antibodies were tested against 8 different isolates, antibodies to each one of these correlated negatively with the expression of group A-like *var* genes suggesting that a single parasite isolate can be used as a surrogate of host immunity (18).

The explanation that the observed positive relationship between *var* gene expression and host age in the first year of life is due to the waning maternal antibodies is further supported by the *var* expression homogeneity data where significant negative and positive correlation with host age and αIE, respectively, was observed. That is, as the maternal antibody wanes, the *var* gene expression homogeneity decreases which is consistent with a previous study that demonstrated that homogeneity of *var* gene expression is lowest in children with severe malaria and low αIE as compared to children with asymptomatic infection and high αIE (32).

The observed rise in parasitemia with declining αIE during the first 6 months of life (Figure 1C) is also supportive of a role for maternal αIE in controlling parasitemia potentially through selection against parasites expressing group A and DC8 *var* genes. These subsets of genes mediate stronger cytoadhesion of IE and sequestration in the organs (13, 18, 20, 33), allowing IE escape removal by the spleen and thus promoting *in vivo* expansion of the parasite population. Maternal αIE targeting group A and DC8 *var* genes are expected to limit parasite expansion through inhibition of IE sequestration, rendering the IE vulnerable to splenic clearance (26, 34–36).

Other factors have been proposed to protect against malaria in infants and may play a role in modifying *var* expression during infancy. Fetal hemoglobin (HbF) has been demonstrated to decline in the first year of life (37). It is possible that the decline of HbF in the first year of life could explain the increasing expression of group A *var* genes over the same period and that this coincidentally mimics the effect of antibodies on parasite gene expression. Hemoglobinopathies such as sickle trait have been proposed to protect against malaria by reducing the amount of *Pf*EMP1 trafficked to the surface of IE (38). There is also evidence that sickle red cells can modify the parasite gene expression at transcriptional level through red cell microRNA that translocate into the parasite (39). This raises the possibility that other abnormal hemoglobin, such as HbF, may as well contribute to protection by reducing *var* gene expression and cytoadhesion (40). HbF has been suggested to work together with maternally acquired antibodies to protect infants from malaria (41) perhaps by impairing cytoadhesion and promoting clearance of IE. This suggestion is consistent with our result showing that expression of group A *var* genes increases over the period when both HbF and maternal antibodies are expected to wane. Dissecting the relative contribution of HbF and maternal αIE on the parasite *var* gene expression during the first year of life warrants further investigation.

In conclusion, we have tested the prediction that, if maternal antibodies play a role in protecting infants against severe malaria, they would impose a selection pressure on the infecting parasite population that is similar to that previously described in children as they gain exposure to *P. falciparum* infection. The results show that parasites infecting infants, show *var* gene expression patterns that are highly similar to those previously described for parasites under immune selection. Future studies that relate αIE levels in cord blood with *var* gene expression patterns during first infection, or longitudinal studies of mother-infant pairs, would be required to provide a definitive link between maternal antibodies and protection against severe malaria in the first months of life.

## Data Availability Statement

The datasets generated for this study can be found in the Harvard Dataverse, URL: https://doi.org/10.7910/DVN/VGRAVH.

## Ethics Statement

The studies involving human participants were reviewed and approved by KEMRI/SERU/3149 Kenya Medical Research Institute. Written informed consent to participate in this study was provided by the participants' legal parents/guardians.

## Author Contributions

PB, KM, CK, and AA contributed to the design and implementation of the research. CK, AA, and MM performed the experiments with contributions from MH, GF, TO, GG, GW, and RR for sequence data and statistical analyses. The manuscript was prepared by CK with contributions from AA, KM, GF, and PB. All authors read and approved the final manuscript.

### Conflict of Interest

The authors declare that the research was conducted in the absence of any commercial or financial relationships that could be construed as a potential conflict of interest.
