# Supplementary material for: Exploring Plasmodium falciparum Var Gene Expression to Assess Host Selection Pressure on Parasites During Infancy
Source: Front Immunol. 2019 Oct 9;10:2328. doi: 10.3389/fimmu.2019.02328 (PMC6798654; doi:10.3389/fimmu.2019.02328)
Supplement: Supplementary file 1 [file Presentation_1.pptx]

## Slide 1
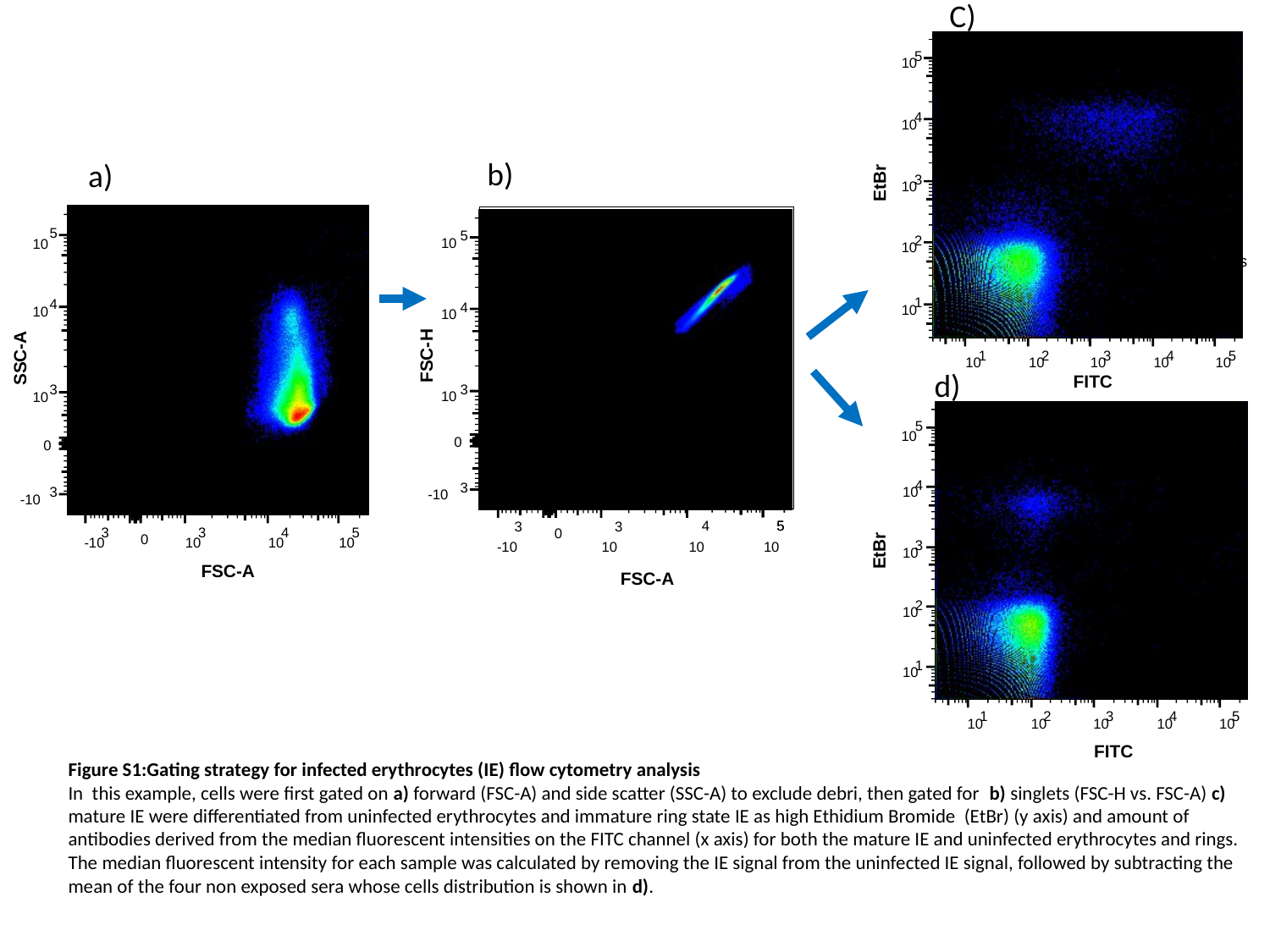

C)
5
10
iRBCs_Trophs
1.51
4
10
3
EtBr
10
2
10
uRBCs+Rings
57.8
1
10
3
4
5
1
2
10
10
10
10
10
FITC
b)
5
10
Single Cells
99.7
4
10
FSC-H
3
10
0
3
3
3
0
-10
10
FSC-A
4
5
5
10
10
-10
a)
5
10
4
10
SSC-A
3
10
0
3
-10
3
3
4
5
0
-10
10
10
10
FSC-A
d)
5
iRBCs_trophs
10
1.55
4
10
EtBr
3
10
2
10
uRBCs+Rings
1
10
63.7
1
2
3
4
5
10
10
10
10
10
FITC
Figure S1:Gating strategy for infected erythrocytes (IE) flow cytometry analysis
In this example, cells were first gated on a) forward (FSC-A) and side scatter (SSC-A) to exclude debri, then gated for b) singlets (FSC-H vs. FSC-A) c) mature IE were differentiated from uninfected erythrocytes and immature ring state IE as high Ethidium Bromide (EtBr) (y axis) and amount of antibodies derived from the median fluorescent intensities on the FITC channel (x axis) for both the mature IE and uninfected erythrocytes and rings. The median fluorescent intensity for each sample was calculated by removing the IE signal from the uninfected IE signal, followed by subtracting the mean of the four non exposed sera whose cells distribution is shown in d).

## Slide 2
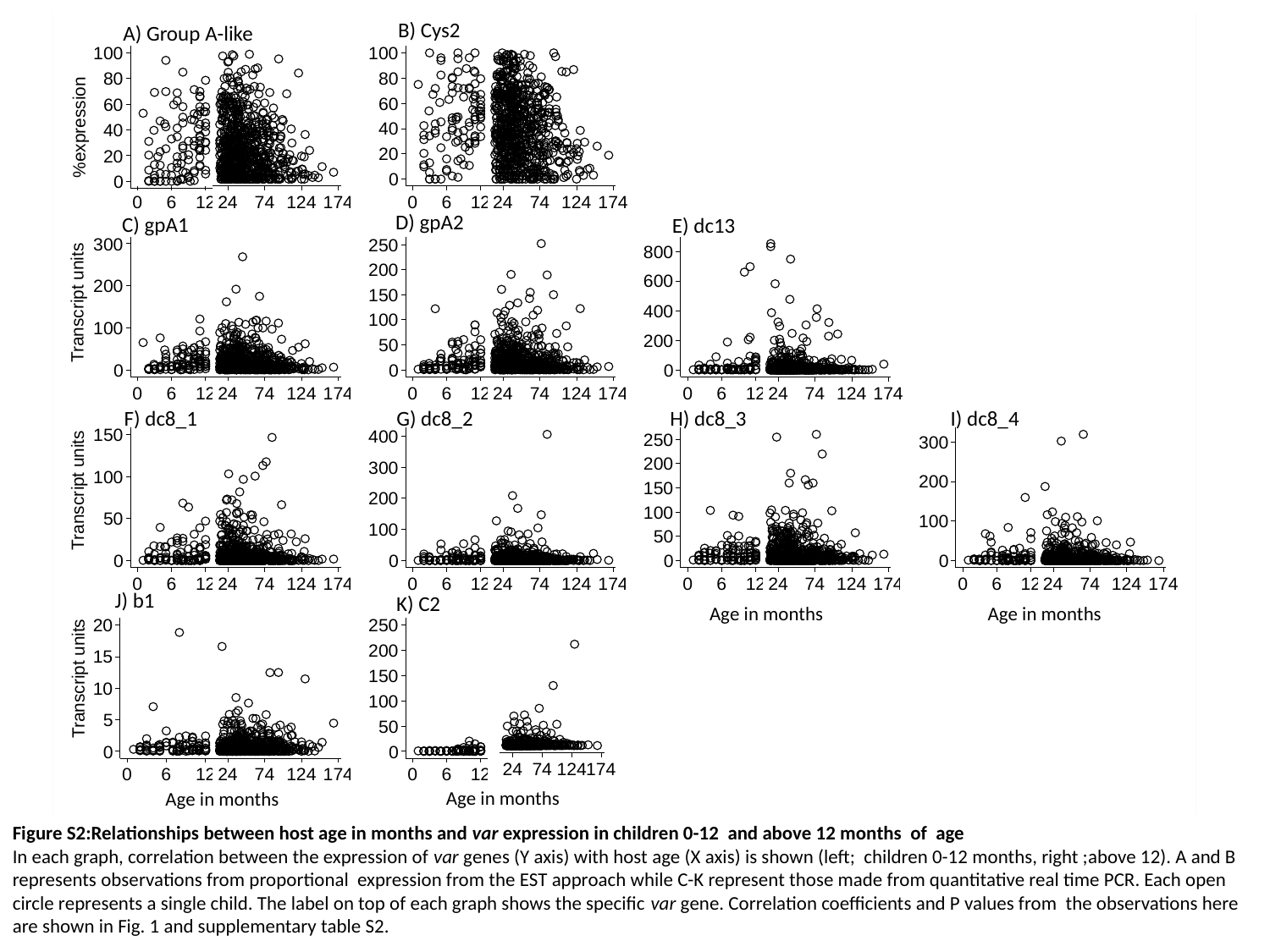

B) Cys2
A) Group A-like
%expression
D) gpA2
C) gpA1
E) dc13
Transcript units
I) dc8_4
G) dc8_2
H) dc8_3
F) dc8_1
Transcript units
J) b1
K) C2
Transcript units
Age in months
Age in months
Age in months
Age in months
Figure S2:Relationships between host age in months and var expression in children 0-12 and above 12 months of age
In each graph, correlation between the expression of var genes (Y axis) with host age (X axis) is shown (left; children 0-12 months, right ;above 12). A and B represents observations from proportional expression from the EST approach while C-K represent those made from quantitative real time PCR. Each open circle represents a single child. The label on top of each graph shows the specific var gene. Correlation coefficients and P values from the observations here are shown in Fig. 1 and supplementary table S2.

## Slide 3
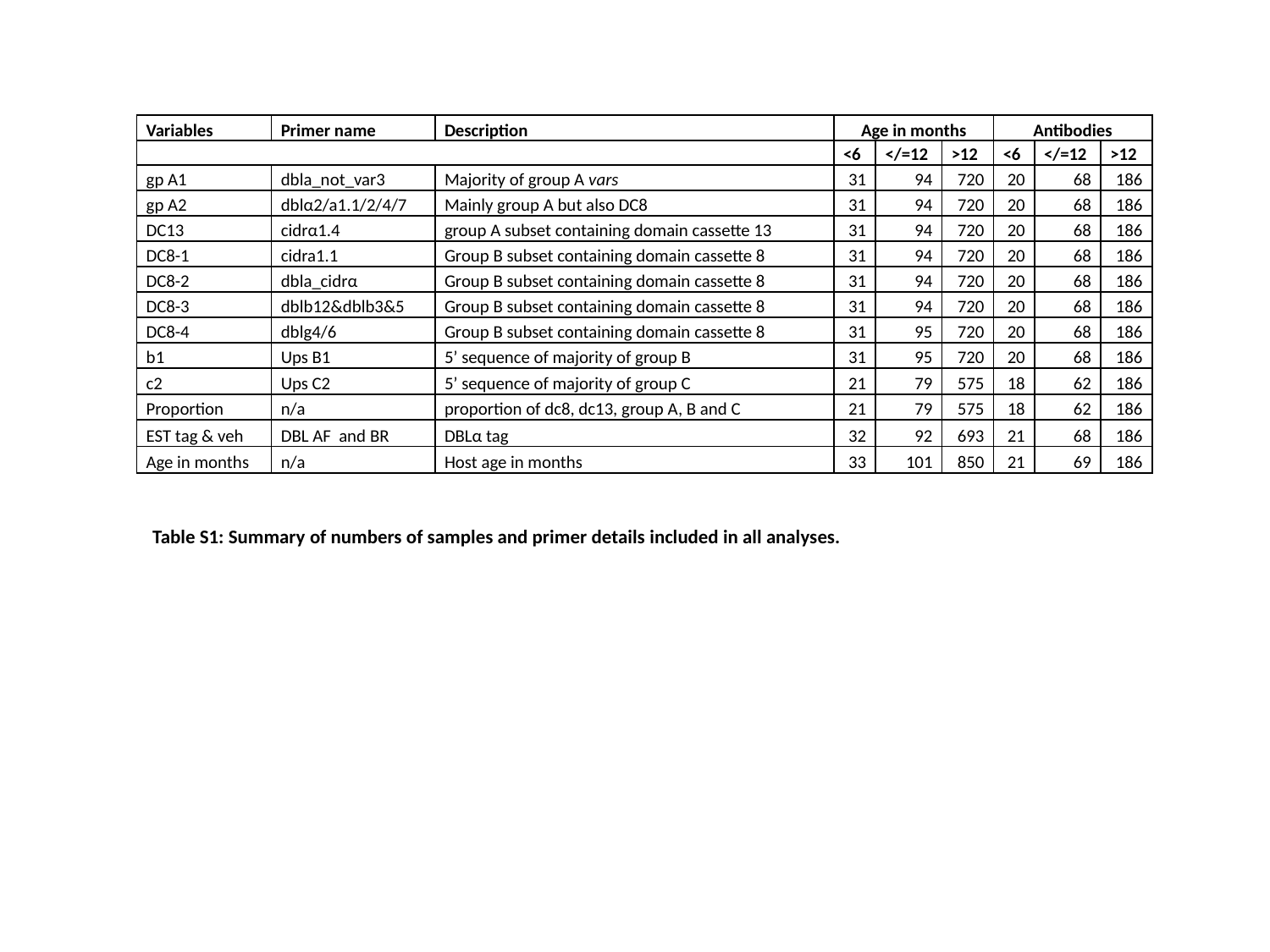

| Variables | Primer name | Description | Age in months | | | Antibodies | | |
| --- | --- | --- | --- | --- | --- | --- | --- | --- |
| | | | <6 | </=12 | >12 | <6 | </=12 | >12 |
| gp A1 | dbla\_not\_var3 | Majority of group A vars | 31 | 94 | 720 | 20 | 68 | 186 |
| gp A2 | dblα2/a1.1/2/4/7 | Mainly group A but also DC8 | 31 | 94 | 720 | 20 | 68 | 186 |
| DC13 | cidrα1.4 | group A subset containing domain cassette 13 | 31 | 94 | 720 | 20 | 68 | 186 |
| DC8-1 | cidra1.1 | Group B subset containing domain cassette 8 | 31 | 94 | 720 | 20 | 68 | 186 |
| DC8-2 | dbla\_cidrα | Group B subset containing domain cassette 8 | 31 | 94 | 720 | 20 | 68 | 186 |
| DC8-3 | dblb12&dblb3&5 | Group B subset containing domain cassette 8 | 31 | 94 | 720 | 20 | 68 | 186 |
| DC8-4 | dblg4/6 | Group B subset containing domain cassette 8 | 31 | 95 | 720 | 20 | 68 | 186 |
| b1 | Ups B1 | 5’ sequence of majority of group B | 31 | 95 | 720 | 20 | 68 | 186 |
| c2 | Ups C2 | 5’ sequence of majority of group C | 21 | 79 | 575 | 18 | 62 | 186 |
| Proportion | n/a | proportion of dc8, dc13, group A, B and C | 21 | 79 | 575 | 18 | 62 | 186 |
| EST tag & veh | DBL AF and BR | DBLα tag | 32 | 92 | 693 | 21 | 68 | 186 |
| Age in months | n/a | Host age in months | 33 | 101 | 850 | 21 | 69 | 186 |
Table S1: Summary of numbers of samples and primer details included in all analyses.
